# FOSL1’s Oncogene Roles in Glioma/Glioma Stem Cells and Tumorigenesis: A Comprehensive Review

**DOI:** 10.3390/ijms25105362

**Published:** 2024-05-14

**Authors:** Azam Khedri, Shanchun Guo, Vanajothi Ramar, BreAnna Hudson, Mingli Liu

**Affiliations:** 1 Department of Microbiology, Biochemistry & Immunology, Morehouse School of Medicine, Atlanta, GA 30310, USA; 2 RCMI Cancer Research Center, Department of Chemistry, New Orleans, LA 70125, USA

**Keywords:** glioblastoma, FOSL1, glioma stem cells, drug resistance, prognosis, biomarker

## Abstract

This review specifically examines the important function of the oncoprotein FOSL1 in the dimeric AP-1 transcription factor, which consists of FOS-related components. FOSL1 is identified as a crucial controller of invasion and metastatic dissemination, making it a potential target for therapeutic treatment in cancer patients. The review offers a thorough examination of the regulatory systems that govern the influence exerted on FOSL1. These include a range of changes that occur throughout the process of transcription and after the translation of proteins. We have discovered that several non-coding RNAs, such as microRNAs (miRNAs) and long non-coding RNAs (lncRNAs), play a significant role in regulating FOSL1 expression by directly interacting with its mRNA transcripts. Moreover, an investigation into the functional aspects of FOSL1 reveals its involvement in apoptosis, proliferation, and migration. This work involves a comprehensive analysis of the complex signaling pathways that support these diverse activities. Furthermore, particular importance is given to the function of FOSL1 in coordinating the activation of several cytokines, such as TGF-beta, and the commencement of IL-6 and VEGF production in tumor-associated macrophages (TAMs) that migrate into the tumor microenvironment. There is a specific emphasis on evaluating the predictive consequences linked to FOSL1. Insights are now emerging on the developing roles of FOSL1 in relation to the processes that drive resistance and reliance on specific treatment methods. Targeting FOSL1 has a strong inhibitory effect on the formation and spread of specific types of cancers. Despite extensive endeavors, no drugs targeting AP-1 or FOSL1 for cancer treatment have been approved for clinical use. Hence, it is imperative to implement innovative approaches and conduct additional verifications.

## 1. Introduction

Glioblastoma multiforme (**GBM**) is an aggressive and malignant form of brain tumor that often recurs despite intensive therapy. This recurrence is often associated with resistance to treatment and can be attributed to various factors, with glioblastoma stem cells (**GSCs**), playing a significant role. GSCs are a small subpopulation of cells within the tumor that exhibit stem-cell-like properties, such as self-renewal and the capacity to differentiate into various cell types found within the tumor. GSCs contribute to the recurrence and resilience of glioblastoma in many ways, such as therapy resistance, heterogeneity, microenvironment interaction, invasion, migration, and tumor regrowth [1]. GSCs possess many markers that distinguish them, such as CD133 (Prominin 1), CD15, CD44, integrin alpha α6, L1CAM, and A2B5 on the cell surface, as well as the cytoplasmic proteins SOX2, Nanog, Olig2, Myc, Musashi1, and nestin [2,3]. CD133 has been associated with stem-cell-like properties in various cancers, including brain tumors; in many studies involving brain cancer stem cells, researchers have utilized CD133 as a cell surface marker to isolate and analyze these cells based on their functional traits. A recent study revealed that four specific transcription factors (SOX2, SALL2, OLIG2, and POU3F2) can induce a conversion of differentiated glioblastoma cells into cells with stem-like qualities [4]. FOSL1 manages the activity of these four factors by binding to their promoter regions, thereby influencing various markers associated with stemness. Additionally, it diminishes the cells’ ability to aggregate by increasing the presence of fibronectin1 FN1 in the extracellular matrix. FOSL1 orchestrates changes in stemness by regulating the behavior of these four transcription factors [4].

FOSL1 is a gene that encodes Fos-related-antigen-1 (FRA-1) consisting of 271 amino acids. FOSL1 is a member of the FOSL1 family belonging to the activator protein-1 (AP-1) transcription factor superfamily. The AP-1 complex results from the dimerization between members of the JUN (c-Jun, JunB, and JunD), FOS (FOS, FOSB, FOSL1, and FOSL2) family members, and other activating transcription factor protein families (ATF and Maf) [5,6]. JUN can form both JUN–JUN homodimers and JUN–FOS heterodimers, while FOS can only form JUN–FOS heterodimers with JUN. The formation of AP-1 dimers is dependent on the basic leucine zipper (bZIP) domain on JUN and FOS. The bZIP domain of FOSL1 consists of two regions: a basic region and a leucine zipper region. Activator Protein 1 (AP-1) constitutes a dimeric transcription factor composed of members of the Fos, Jun, Maf, and ATF multigene families. These AP-1 dimers bind to different types of palindromic sequences depending on their compositions [7,8]. The two main types of AP-1 sites are TPA-responsive elements (TREs) and cyclic AMP response elements (CREs). TREs are palindromic sequences that are responsive to the phorbol ester 12-O-tetradecanoylphorbol-13-acetate (TPA) and are commonly found in the promoters of genes involved in cell proliferation, differentiation, and survival. On the other hand, CREs are palindromic sequences that respond to cyclic AMP (cAMP) and play a crucial role in regulating genes associated with metabolism, inflammation, and stress response [9,10]. 

FOSL1 has been found to exhibit high expression levels and demonstrated an oncogene in numerous tumor types including thyroid tumors, gastric cancer (GC) [11], breast cancer, hepatocellular carcinoma (HCC) [12], head and neck squamous cell carcinoma (HNSCC) [13], nephroblastoma, and laryngeal squamous cell carcinoma [10,14]. In a single study, FOSL1 demonstrated tumor suppressor activity in cervical carcinoma [15]. The scientists demonstrated that FOSL1 suppressed the growth of cervical cancer cells by inducing programmed cell death and altering the distribution of cells in the cell cycle. In addition, FOSL1 increased the expression of STAT1 and influenced the activity of the p53 signaling pathway in cervical cancer cells.

In this paper, we provide a comprehensive summary of recent studies focusing on the transcriptional, epigenetic regulation, and signal transduction mechanism of FOSL1. We explore its significant role in various aspects of cancer biology, including cell proliferation, survival, epithelial-mesenchymal transition (EMT), metastasis, prognosis, and drug resistance in glioma and other tumor types. Moreover, we discuss the critical implications of FOSL1 in the diagnosis and treatment of tumors with a special emphasis on glioblastoma, emphasizing its relevance in targeted therapy approaches.

## 2. Regulation of FOSL1

### 2.1. Transcriptional Regulation

The expression and activity of FOSL1 can be regulated by various factors, including extracellular signals, transcriptional and post-transcriptional mechanisms, and epigenetic modifications [16]. The expression of the FOSL1 gene is controlled by several transcription factors that bind to either the promoter area (AP-1, beta-catenin, TWIST1, SNAIL1, Elk, SRF, ATF, and CREB) or the intronic enhancer (AP-1, MYC, TWIST1, and SNAIL1). The positive autoregulation of FOSL1 is mediated by multiple binding sites of the AP-1 transcription factor. This positive feedback loop is further amplified by the posttranslational accumulation of FOSL1. The BRD4 epigenetic reader, which is associated with the enhancer, plays a key role in the recruitment of p-TEFb, which in turn phosphorylates the Carboxy-Terminal Domain (CTD) of RNA polymerase II (RNAPII). This triggers transcriptional elongation by releasing the RNAPII paused on the *FOSL1* promoter [16,17]. Notably, the FOSL1 intronic enhancer is a component of a significantly bigger Super Enhancer (SE) region, which has been discovered through comprehensive genome-wide investigations in GBM [18,19], pancreatic, and colorectal cancer cells. SEs are groups of enhancers that strongly activate the transcription of genes crucial for cell identity and disease phenotype [17]. Our earlier work indicated that TRPM7 controls the stemness of glioma cells by influencing STAT3 [20]. In order to better understand how TRPM7 promotes the transcription of the FOSL1 gene to contribute to glioma stemness, we created a FOSL1 promoter and its GAS mutants. We then conducted luciferase reporter experiments and ChIP-qPCR to gather further information. The collective findings indicate that TRPM7 triggers the activation of FOSL1 transcription, which is facilitated by the activity of STAT3. This process has been identified as crucial in maintaining the stemness of glioma cells [21]. 

### 2.2. Non-Coding RNAs Regulate FOSL1

The two main types of regulatory non-coding RNAs (ncRNAs) are long non-coding RNAs (lncRNA, >200 nts) and microRNAs (miRNAs, <30 nts). The function of miRNAs is to regulate gene expression by binding to the untranslated region (3′-UTR) of the target mRNAs. On the other hand, lncRNA can act as competing endogenous RNAs (ceRNAs) and sponge miRNAs subsequently reduce the function of miRNAs. The cross-talk between lncRNAs, miRNAs, and mRNAs has a pivotal role in the pathogenesis of malignancy [22,23]. Recently, there has been a growing interest in investigating the posttranscriptional regulation of FOSL1 by ncRNAs. Studies have revealed that several non-coding RNAs, including miRNAs and lncRNAs, are involved in regulating FOSL1 expression by targeting its mRNA transcripts [24,25,26]. 

#### 2.2.1. miRNAs and FOSL1

**Figure 1:**
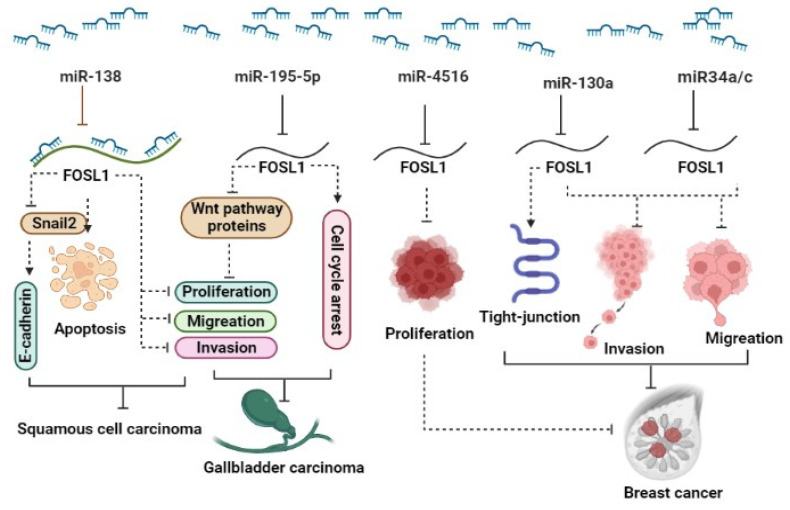
A schematic representation illustrates the role of miRNAs in the regulation of FOSL1. Different miRNAs act to modulate (inhibit) the expression levels of FOSL1, thus exerting an influence on various aspects of cancer behavior. Through the downregulation of FOSL1, these miRNAs influence the Wnt signaling pathway, apoptosis, cell proliferation, cell cycle, invasion, and migration, ultimately resulting in the reduction in tumor growth across a range of types.

The ectopic expression of *FOSL1* was reported in several types of malignancy. Recent studies have shown that several miRNAs target *FOSL1* and regulate its expression, leading to the modulation of various cellular functions. It has been reported that the downregulation of miRNAs can result in increased expression of FOSL1 in various cancers [27,28,29]. (***1***) Breast cancer (BC): The expression levels of miR34a/c in patients significantly decreased in patients with metastatic breast tumors as well as in the metastatic cancerous cell lines MDA-MB-231 and Hs578T. In vitro and in vivo studies reveal that ectopic expression of the two miR34a/c suppressed the invasion and migration of cancerous breast cells. Yang et al. reported that overexpression of miR34a/c can directly bind to 3′ UTR-FOSL1, consequently inhibiting its expression at both mRNA and protein levels [30]. It has been reported that elevated levels of FOSL1 in triple-negative breast cancer (TNBC) patients are associated with poor survival. miR-4516 target genes such as FOSL1 are involved in the regulation of proliferation [31]. They demonstrated a significant downregulation of miR-4516 expression in patients with invasive ductal carcinoma [31]. Furthermore, their findings revealed that the overexpression of miR-4516 significantly inhibited the proliferation of TNBC cells by targeting the *FOSL1* gene [31]. In addition, ectopic expression of miR-130a targets FOSL1 and suppresses invasion and migration in invasive breast cancer Hs578T and MDA-MB-231 cell lines. The overexpression of miR-130a led to an increase in ZO-1 expression, a tight-junction protein, through the downregulation of FOSL1 [29]. miR-19a-3p has also been implicated in the regulation of FOSL1 in tumor-associated macrophages (TAMs). The downregulation of miR-19a-3p results in the accumulation of FOSL1 in TAMs, which in turn promotes the invasion and metastasis of breast cancer cells [32]. (***2***) Colorectal cancer (CRC): The downregulation of miR34a/c and miR-15/16-family member miR-497 has been observed in CRC. This downregulation plays a significant role in promoting neoplastic cell invasion and EMT driven by FOSL1. These miRNAs are known to target FOSL1, leading to its upregulation and subsequently, the activation of genes involved in EMT and metastasis [33,34]. (***3***) Gallbladder carcinoma (GC): In gallbladder cancer, miR-195-5p has been identified as being downregulated, and this downregulation promotes cell proliferation, migration, and invasion. It achieves this effect by targeting FOSL1, which, in turn, activates the Wnt/β-catenin signaling pathway [28]. In vitro investigations revealed that overexpression of miR-195-5p in NOZ and GBC-SD gallbladder carcinoma cell lines not only suppressed proliferation, invasion, and migration but also induced cell cycle arrest in the G0/G1 phase. miR-195-5p exerts this function via targeting FOSL1 and inhibiting the expression of proteins including β-catenin, CCND1, and c-Myc that are involved in the Wnt signaling pathway [28]. (***4***) Squamous cell carcinoma (SCC): miR-138 is a tumor suppressor that regulates the expression of various oncogenes, including FOSL1. In squamous cell carcinoma, the down-regulation of FOSL1 by microRNA-138 may inhibit cell proliferation and invasion [35]. In this paper, Yi et al. reported that miR-138 binds directly to the 3′-UTR of *FOSL1*, leading to a decrease in its expression. Moreover, miR-138 was found to indirectly regulate FOSL1 target genes, including Snail2. Subsequently, Snail2 was observed to have an effect on E-cadherin expression. They also discovered that miR-138 can bind not only to the 3′-UTR of FOSL1 but also to the 5′-UTR and coding region of *FOSL1*. In summary, the miR-138/FOSL1/Snail2 axis plays a pivotal role in the progression of squamous cell carcinoma [35]. (***5***) Glioma: FOSL1, functioning as a transcriptional factor, has exhibited upregulation in several tumors, including glioma. Rong et al. demonstrated that radiotherapy treatment leads to the induction of apoptosis and inhibition of cell viability in the glioma cancer stem cell (CSC) lines CSC-U-251MG and CSC-A172. After radiotherapy, the expression of FOSL1, miR-27a-5p, and Bcl-2 remarkably decreased, while Bax and cleaved caspase-3 increased. Using bioinformatics tools, they have demonstrated that FOSL1 exhibits a significant signal in the promoter region of miR-27a-5p. Remarkably, the silencing of FOSL1 sensitizes glioma cancer stem cells through the downregulation of miR-27a-5p, whereas the ectopic expression of FOSL1 reduces the effectiveness of radiotherapy on apoptosis and cell viability by modulating miR-27a-5p [27] (Figure 1).

#### 2.2.2. LncRNA and FOSL1

**Figure 2:**
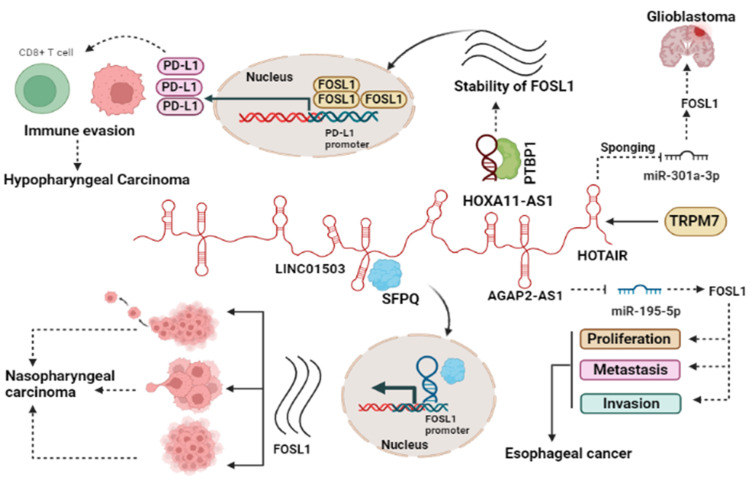
lncRNAs regulate the function of FOSL1 through various mechanisms. Oncogenic lncRNAs act as miRNA sponge, leading to increased FOSL1 ectopic expression as a target of the miRNA. Additionally, lncRNAs interact with specific proteins to enhance the stability of FOSL1, participating in immune regulation, or translocate to the nucleus to influence gene expression. The interplay between lncRNAs and FOSL1 contributes to heightened cancer cell growth by impacting immune evasion, migration, invasion, and cell proliferation.

Several studies reported the involvement of lncRNAs such as HOTAIR [26], AGAP2-AS1, LINC01503, and HOXA11-AS1 in the regulation of FOSL1 in cancer [24,25,36]. Our investigation revealed that the lncRNA HOTAIR functions as a competitive endogenous RNA (ceRNA) by sequestering miR-301a-3p and subsequently controlling the expression of FOSL1 indirectly [26]. (***1***) GBM: TRPM7 is one of the members of channel TRP superfamily that has a pivotal role in malignancy. Our group reported that TRPM7 serves as a positive regulator of lncRNA HOTAIR and contributes to gliomagenesis. In glioblastoma, HOTAIR accelerates tumor progression by acting as a sponge for miR-301a-3p, leading to an increase in the expression of FOSL1, a key player in glioma progression. Silencing of HOTAIR results in increased miR-301a-3p expression, leading to the suppression of FOSL1 and subsequently reducing cell proliferation, invasion, and migration [26]. (***2***) In esophageal cancer (EC), AGAP2-AS1 sponges miR-195-5p and subsequently upregulates FOSL1, which in turn increases the proliferation of cancerous cells, metastasis, and invasion. The knockdown of AGAP2-AS1 leads to the promotion of miR-195-5p expression, which, in turn, inhibits FOSL1 ectopic expression, subsequently impeding tumor progression. Moreover, this knockdown also increases apoptosis and causes cell cycle arrest in the G0/G1 phase [25]. (***3***) In nasopharyngeal carcinoma (NPC), LINC01503 recruits splicing factor proline- and glutamine-rich (SFPQ) protein and colocalizes in the nucleus. They jointly bind to the promoter region of *FOSL1* genes, thereby promoting their expression. It can be concluded that the LINC01503/SFPQ/FOSL1 axis is involved in different aspects of NPC development including cell proliferation, migration, invasion, tumor growth, and metastasis [24]. HOXA11-AS1 lncRNA plays a critical role in metastasis in hypopharyngeal squamous cell carcinoma (HSCC). It achieves this function by regulating the mRNA stability of *FOSL1* through its binding to PTBP1, which acts as a key regulator of tumorigenesis. On the other hand, FOSL1 binds to the PD-L1 promoter, leading to increased PD-L1 expression and promoting immune escape. Taken together, the interaction between HOXA11-AS1, FOSL1, PTBP1, and PD-L1 forms a crucial axis that collectively enhances proliferation, metastasis, immune escape, and tumor growth [36] (Figure 2).

### 2.3. Post-Translational Regulation

Post-translational modifications are essential for regulating the accumulation of FOSL1, in addition to transcriptional control. These alterations impact the duration of the protein’s existence and encompass activities such as phosphorylation, acetylation, and ubiquitination. The phosphorylation of FOSL1 by various kinases, such as ERK and JNK, can stabilize or destabilize the protein depending on the context of the signaling pathways involved. Acetylation of FOSL1 can increase its stability and transcriptional activity, while ubiquitination can target FOSL1 for degradation by the proteasome [19,37,38]. (***1***) FOSL1 Phosphorylation: FOSL1 has several phosphorylation sites at serine and threonine residues. ERK2 and Rsk1 kinases phosphorylate serine residues at positions S252 and S265, respectively, while PKC-theta phosphorylates threonine residues at positions T223 and T230 [6,39]. Phosphorylation at these specific sites protects FRA-1 from degradation in proteasomes and consequently stabilizes its expression levels. Furthermore, phosphorylation of threonine residues also enhances FOSL1’s biological activity, leading to increased transcriptional activation of downstream target genes [40,41]. For example, when Thr231 (T223) is substituted with Asp, it generates a dominant negative variant of FOSL1. This mutated form of FOSL1 loses its ability to activate FOSL1 target genes, including Mmp1 [41,42]. (***2***) FOSL1 acetylation/deacetylation: acetylation can also regulate the function of FOSL1. The IL6/STAT3 axis is involved in the deacetylation of FOSL1 lysine-116 residue by the deacetylase HDAC6 in CRCs. This modification results in the cancer cells acquiring stemness properties, which represents a critical step in the development and progression of cancer [43]. The latest research findings indicate that removing the acetyl group from FOSL1 at the Lys-16 position in its DNA binding domain results in an enhancement of FOSL1’s ability to activate transcription [21].

## 3. The General Roles of FOSL1 in Glioma and Other Tumor Types 

FOSL1plays a crucial role in EMT, which is essential for cancer cell migration and metastasis. Additionally, FOSL1 has been implicated in the regulation of genes involved in angiogenesis, immune evasion, and resistance to chemotherapy and targeted therapies. Hence, FOSL1 emerges as a key transcription factor contributing to various aspects of tumor development and progression in glioma and other tumor types (Table 1). An early study evaluated the gene expression profiles resulting from the inducible expression of either wild-type EGFR or EGFRvIII, at comparable levels, in a U251-MG glioma cell line [44]. The authors demonstrated that EGFRvIII expression led to the selective up-regulation of a limited set of genes. Notably, all of these genes, such as TGFA, HB-EGF, EPHA2, IL8, MAP4K4, EMP1, DUSP6, as well as FOSL1, have an impact on signaling pathways that are recognized to play a significant role in the development of cancer. Halatsch et al. identified FOSL1 as one of the five genes (BDNF, CARD6, FOSL1, HSPA9B, and MYC) involved in antiapoptotic pathways which were unexpectedly found to be associated with mediating the cellular response of human GBMs to erlotinib treatment [45]. Since then, FOSL1 has been extensively studied by various groups including our own, specifically for its significant role in promoting proneural (PN) to mesenchymal (MSN) states in gliomagenesis. It achieves this through pathways involving NF1 [46] and the UBC9/CYD/NF-κB axis [47] contributing to its widespread oncogenic roles. Even when EGFR and MET were inhibited, the suppression of FOSL1 phosphorylation was temporary, rebounding within 48 h. Consequently, the rebound led to increased transcription levels of five FOSL1 target genes, namely SPRY2, MMP2, JUN, PLAUR, MMP1, and IL-6, contributing to resistance against kinase inhibitors [48,49,50,51,52,53,54]. FOSL1 serves as a clinical prognostic marker for glioma [21,26,55]. 

### 3.1. The Role of FOSL1 in Apoptosis and Proliferation and Its Associated Signaling Pathways

Many tumor types have an overexpression of FOSL1. CRISPR-induced *FOSL1* transcription led to FOSL1 overexpression, causing a decrease in cell adhesion to fibronectin and collagen, consequently impacting cell cycle progression [68]. Its overexpression contributes to tumor progression by regulating several cell signaling pathways. In GC, FOSL1 has been shown to inhibit apoptosis and promote cell proliferation. The upregulation of FOSL1 in GC cells leads to the activation of the PI3K/AKT pathway and the upregulation of MDM2. MDM2, in turn, inhibits the activity of the p53 tumor suppressor gene, which plays a key role in regulating apoptosis and cell cycle arrest. Taken together, the overexpression of FOSL1 in GC cells leads to the inhibition of apoptosis and the promotion of cell proliferation by activating the PI3K/AKT pathway and inhibiting the activity of the p53 tumor suppressor gene [63]. Silencing FOSL1 leads to the suppression of the ERK/AP-1 signaling pathway, effectively inhibiting osteosarcoma (OS) cell proliferation, invasion, and migration [69]. In nasopharyngeal carcinoma cells (CNE1), there is an upregulation of mitogen and stress-activated kinase 1 (MSK1) expression. Furthermore, the induction of EBV latent membrane protein 1 (LMP1) leads to an increase in the phosphorylation of histone H3 at Ser10. These mechanisms are believed to mediate the effects of FOSL1 on cell proliferation and apoptosis inhibition in these cancer cells [64]. The FOSL1 gene is commonly found to be overexpressed in thyroid cell transformations. When FOSL1 is knocked out, cell proliferation is arrested, leading to apoptosis. Moreover, the majority of cells accumulate in the G2 phase, while a few undergo abnormal cell division. FOSL1 binds to the previously unidentified AP-1 site and CRE to the cyclin A promoter during the cell cycle, promoting cell proliferation [70]. Additionally, FOSL1 induces the expression of Jun B, which interacts with the cyclin A promoter, subsequently enhancing cell growth and survival in RAS-transformed thyroid cells [71]. Therefore, FOSL1 is a critical regulator of cell cycle progression. Psoralen has been discovered to display antiproliferative effects on breast cancer cells due to its ability to increase the expression of Axin2 and decrease the expression of FOSL1, suggesting that psoralen’s mechanism of action in breast cancer involves the regulation of the cell cycle by modulating the expression of Axin2 and FOSL1 [72]. Breast cancer cells, specifically MCF-7 and MDA-MB-231, have high FOSL1 levels, enhancing their proliferative and metastatic ability [73,74]. 

The effects of FOSL1 on apoptosis are contradictory in different types of cancer. (***1***) Studies have shown that in cervical cancer, the expression of FOSL1 is reduced in tumor cells compared to normal tissue. Consequently, downregulation of MDM2 and consequent p53 accumulation favors apoptosis, indicating that FOSL1 functions as a pro-apoptotic factor [15,75]. The reasons for these inconsistencies remain unclear. (***2***) FOSL1 is significantly upregulated in lung cancer. Studies have shown that lung cancer cells with elevated FOSL1 ectopic expression demonstrated reduced apoptosis rates compared to control cells. The decrease in apoptosis is strongly correlated with a reduction in mitochondrial membrane potential (Δψm) and an elevation in intracellular levels of reactive oxygen species (ROS) and calcium ions (Ca^2+^). On the other hand, when FOSL1 is overexpressed, it causes an elevation in the electrical potential across the mitochondrial membrane, a reduction in the levels of ROS and Ca^2+^ inside the cell, and finally results in the prevention of apoptosis in lung cancer cells [76]. Yet, it is crucial to approach the data explanation cautiously as the study solely relied on either the Hela cell line [15] or H460 lung cancer cell line [76] and included data from only 20 [15] or 10 [76] glioma patients, respectively. Therefore, deriving a broad conclusion about all cervical cancers from this paper might be challenging. Studying the pathways implicated in cancer development is of great value as it helps identify potential targets for therapy, which is crucial in effectively managing medical conditions.

### 3.2. The Role of FOSL1 in Migration and Its Associated Signaling Pathways

FOSL1 is responsible for inducing changes in the structure and arrangement of the cytoskeleton, loss of polarization in epithelial cells, increased ability to move, and invasion of tumor cells. These changes indicate different degrees of mesenchymal transformation that are dependent on the context, ranging from partial to complete EMT [77]. The overexpression of FOSL1 in immortalized epithelial cells results in the inhibition of epithelial indicators, such as cadherin E (Cdh1), and the activation of endothelial markers, such as vimentin (Vim), Ascdh2, fibronectin (Fn1), Cdh3, S100a4, and Spp1. In addition, overexpression of FOSL1 results in an increase in the expression of the genes that encode integrins 5 and 1 (Itgba5 and Itgb1, respectively) [78]. Cells with silenced FOSL1 exhibit the opposite effects compared to cells with FOSL1 overexpression. Silenced FOSL1 leads to the downregulation of genes associated with the mesenchymal phenotype, including Snail, Slug/Snai2, Zeb1, and Zeb2. These genes are transcription factors that promote the process of EMT [78,79].

#### 3.2.1. CRC

The levels of FOSL1 ectopic expression are correlated with the degree of local invasion, lymph node involvement, and liver metastases in patients with colorectal cancer (CRC). Interleukin-6 (IL-6), a cytokine that promotes inflammation, plays a critical role in the growth and advancement of CRC. Activation of STAT3 in CRC cell lines, triggered by IL-6-mediated EMT, stimulates the elevation of FOSL1 gene expression. Consequently, this raises the production of EMT-promoting proteins (ZEB1, Snail, Slug, MMP-2, and MMP-9), resulting in heightened invasiveness of CRC cells. Therefore, the IL-6/STAT3/FOSL1 signaling pathway initiates EMT, therefore enhancing the ability of CRC cells to invade surrounding tissues [80]. In another study, it was discovered that FOSL1 downregulates the expression of FBXL2, an E3 ligase complex known for its role in inhibiting tumorigenesis by ubiquitinating Aurora B. The downregulation of FBXL2 is believed to be facilitated by FOSL1 through the regulation of the transcriptional activities of Smurf1. These events are thought to be mediated by the Wnt/β-catenin signaling pathway, implying that targeting FOSL1 using shRNA could be a promising therapeutic approach for treating CRC [81]. MicroRNA-34a (miR-34a) is a gene that suppresses tumor growth and is activated by p53. It is essential in preventing the movement and spread of colon cancer cells. This is accomplished by increasing the activity of miR-34a through p53 DNA, resulting in the decrease in FOSL1 abnormal expression, which subsequently inhibits the expression of matrix metalloproteinases, MMP-1 [33]. The stability of FOSL1 in CRC is increased by the action of ubiquitin-specific protease 21 (USP21), which removes ubiquitin molecules from FOSL1, leading to enhanced expression of genes targeted by FOSL1. Additionally, USP21 plays a role in controlling the migration and invasion activities of CRCs through its effect on FOSL1 [60]. The overexpression of FOSL1 may regulate vimentin during the EMT induced by Ha-RAS and contribute to colon cell migration. These findings suggest that in colon cells, the upregulation of proteins such as FOSL1 and vimentin may play a role in inducing epithelial-mesenchymal transition through Ha-RAS oncogenic signaling [82]. PDAP1 plays a role in both mucosal restitution and carcinogenesis in colitis-associated cancer. The upregulation of PDAP1, driven by c-Myc, promotes the proliferation, migration, invasion, and metastasis of CRC cells through the EGFR-MAPK-FOSL1 signaling axis. Therefore, inhibiting PDAP1 may be beneficial for patients with CRC who have high levels of PDAP1 expression [62].

#### 3.2.2. Breast Cancer

FOSL1 has the potential to undergo phosphorylation by various kinases, including mitogen-activated protein kinase (MAPK), protein kinase C (PKC), cAMP-dependent kinase (PKA), and cyclin-dependent kinase 1-cdc2 (CDC2). The continuous stimulation of the MAPK/extracellular signal-regulated kinase (ERK) signaling pathway triggers the migration of breast cancer cells [40]. MLK3 expression induced a significant increase in the oncogenic transcription factor FOSL1, along with elevated levels of MMP-1 and MMP-9, in non-metastatic ER+ BC cells. This increase in invasion was found to be dependent on FOSL1, as silencing FOSL1 reversed the effect of MLK3. In contrast, high levels of FOSL1 in metastatic TNBC were reduced upon MLK3 depletion through gene silencing or CRISPR/Cas9n editing, and both MMP-1 and MMP-9 expression were reduced by inhibiting MLK3 or using an MLK inhibitor [56]. SPAK STE20-related proline/alanine-rich kinase SPAK and ERK1/2 participate in the stabilization of FOSL1 induced by PKCθ, but their involvement varies among different cell lines. The ERK1/2 pathway primarily contributes to the accumulation of FRA-1/FOSL1 in ER-MDA-MB-231 cells, whereas in ER-BT549 cells, SPAK signaling is the main mechanism responsible for the accumulation of FRA-1/FOSL1. PKCθ signaling is a crucial regulator of Fra-1/FOSL1 accumulation in ER-negative breast cancer cells. Furthermore, Fra-1/FOSL1 is essential for PKCθ-induced cell migration and invasion. Taken together, these results indicate that PKCθ may play a role in the progression of certain breast cancers and could potentially serve as a novel therapeutic target [56,57]. FOSL1 plays a role in regulating the expression of High Mobility Group A1(HMGA1) mRNA at the transcriptional level by binding to enhancer elements in breast cancer cells. This promotes the invasion and migration of triple-negative breast cancer cells through RNA polymerase II recruitment [58]. FOSL1 also has other signaling pathways that induce invasion and migration of breast cancer cells, such as the increased expression of integrins αVβ3 and uPAR activating the FAK-SRC-ERK2 signal, leading to increased phosphorylation and activation of FOSL1 [83]. 

#### 3.2.3. Other Cancers

**Figure 3:**
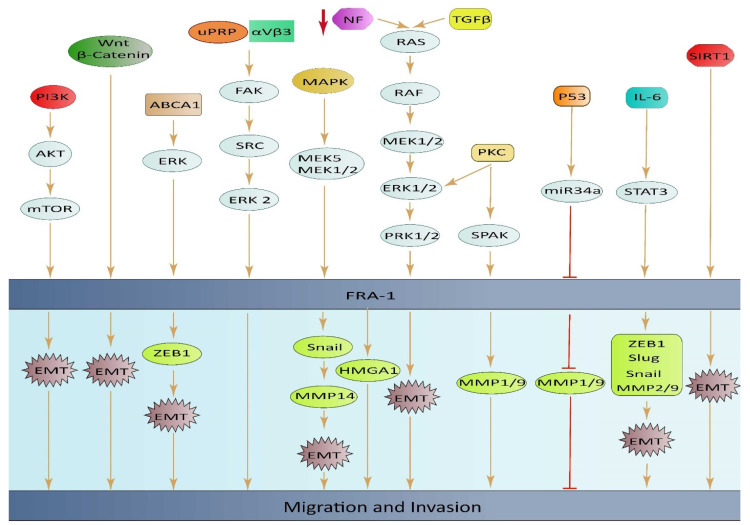
An illustrative diagram depicting the impact of elevated FOSL1 ectopic expression on tumor cell invasion and migration, along with the underlying mechanisms of signal transduction.

ABCA1 plays a role in regulating EMT through the ERK/FOSL1/ZEB1 pathway and has been identified as a new predictor of response to thyroid cancer with lung metastasis. These discoveries highlight the potential of ABCA1 as an important oncogene and a promising therapeutic target for the treatment of thyroid cancer (TC) with lung metastasis [67]. High expression of FOSL1 was found to be significantly linked to positive lymphatic invasion in GC. This finding suggests that FOSL1 might play a crucial part in the migration and invasion of GC cells, but more research is required to confirm this hypothesis [11]. The loss of the neurofibromatosis type 1 gene (NF1) leads to an increase in RAS/MAPK activity, causing changes in the expression of FOSL1. Deletion of FOSL1 prompts a change from a mesenchymal to a proneural transcriptional signature, and results in a reduction in both stemness and tumor growth. The up-regulation of FOSL1 ectopic expression and induction of the EMT program in glioma cells can be attributed to the activation of Wnt/β-catenin signaling. This signaling pathway activates the transcription of FOSL1 by direct binding of β-catenin/Tcf/Lef transcriptional complex to the Wnt response element (WRE) situated in the proximal region of the *FOSL1* promoter [84] (Figure 3). 

### 3.3. The Involvement of FOSL1 in the Tumor Microenvironment

The interaction between TAMs and neoplastic cells within the tumor microenvironment (TME) including stromal cells, tumor cells, fibroblasts, endothelial cells, cytokines, and others, plays a critical role in promoting tumor cell invasion and progression [85,86]. The role of macrophages in cancer is not completely clear, as the M1 phenotype is associated with the ability to kill tumors, while the M2 phenotype of TAMs has a tumor-promoting effect. Interactions between mouse breast tumor cells and TAMs can change the tumor TME, causing an increase in the expression of FOSL1. The function of the *FOSL1* proto-oncogene extends to regulating the expression of IL-6 in macrophages, leading to the development of M2d macrophages [87]. FOSL1 triggers the activation of the IL-6/JAK/Stat3 signaling pathway, resulting in a malignant switch in breast tumor cells that leads to increased release of pro-angiogenic factors such as MMP-9, VEGF, and TGF-β from the tumor cells. Consequently, the activated transcription factor FOSL1 and the IL-6/JAK/Stat3 signaling pathway play a significant role in altering the TME [59].

FOSL1 is highly concentrated in cancer-associated fibroblasts (CAFs) obtained from colorectal cancer (CRC) tissues. Moreover, FOSL1 can be transferred from CAFs to CRC cells via exosomes, which leads to increased CRC cell proliferation and resistance to oxaliplatin. In addition, FOSL1 promotes the expression of integrin β4 (ITGB4) by binding directly to its promoter region [88]. Tumor-initiating cells (T-ICs) in liver cancer are influenced by factors from the TME, just like normal stem cells. Since liver cancer often develops in the context of cirrhosis, which involves activated fibroblasts, researchers have widely investigated if CAFs regulate T-ICs in the liver. The presence of a-SMA (+), CAFs are linked to poor clinical outcomes. CAFs produce HGF, which activates FOSL1 in T-ICs through the Erk1,2 pathway. Further analysis revealed that HEY1 is a direct downstream mediator of FOSL1. In a mouse model of liver cancer, HGF-induced FOSL1 activation is associated with fibrosis-related HCC development. Therefore, targeting the CAF-derived, HGF-mediated c-Met/FOSL1/HEY1 pathway could be a promising therapeutic strategy for treating HCC [89]. miR-19a-3p, responsible for regulating TAMs within the breast TME, could influence the phenotype of TAMs by targeting the *FOSL1* gene and other genes within its downstream signaling pathways. The decreased expression of miR-19a-3p in TAMs is likely caused by TME induction, which encourages the shift from M1 to M2 and subsequently enhances the migration and invasion of breast cancer cells [32].

### 3.4. Cancer Stem Cells 

Cancer stem-like cells possess the ability to self-renew and play significant roles in cancer growth, treatment resistance, and metastasis. Among these cells, glioblastoma stem-like cells have been the subject of the most extensive research.

FOSL1 can transform mature cancer cells into stem-like cells that can propagate the tumor by controlling transcription factors related to stemness [90]. FOSL1 regulates SOX2, SALL2, OLIG2, and POU3F2 by binding to their promoter regions and influences a number of stemness indicators [4]. It also reduces the ability of cells to form aggregates by increasing FN1 in the extracellular matrix. FOSL1 changes stemness by regulating these four transcription factors [4].

The activation of the EMT program during malignant progression can lead to the enrichment of cancer stem cells (CSCs). Inhibiting protein kinase C a (PKCa) can specifically target CSCs with little effect on non-CSCs. The conversion of non-stem cells into CSCs involves a change from EGFR to PDGFR signaling and triggers PKCa-dependent activation of FOSL1. An AP-1 molecular switch has been identified where c-FOS and FOSL1 are used differently in non-CSCs and CSCs. PKCa and FOSL1 ectopic expression is linked to aggressive triple-negative breast cancers, and reducing FOSL1 levels leads to a mesenchymal–epithelial transition. Identifying molecular features that shift between cell states can be used to target critical signaling components in CSCs [91]. The transition from proneural (PN) to mesenchymal (MES) in glioblastoma stem cells (GSCs) is an important shift. FOSL1 plays a key role in regulating this transition. FOSL1 is mainly expressed in the MES subtype of GSCs and not in the PN subtype. Knocking down FOSL1 ectopic expression in MES GSCs results in the loss of MES characteristics and tumorigenicity, while overexpression of FOSL1 in PN GSCs promotes the PN to MES transition and maintains MES characteristics.

FOSL1 also facilitates the induction of the transition to the mesenchymal state by ionizing radiation (IR), leading to an increase in radio resistance in PN GSCs. Blocking FOSL1 can improve the effectiveness of radiotherapy against tumors by preventing the PMT triggered by irradiation. Mechanistically, through the modulation of the IKKb-IkBa-NF-kB signaling pathway, FOSL1 can induce the process of PMT. The introduction of FOSL1 in PN GSCs leads to multiple processes such as the phosphorylation of IKKb on Ser181, degradation of IkBa, and relocation of NF-kB p65 to the nucleus [47]. In the presence of inflammation and IL-6 secretion in the microenvironment, the activation of the STAT3 pathway occurs. This activation, through transcriptional (promoter binding) and post-translational (K116 deacetylation) upregulation of FOSL1/FOSL1, promotes the stemness and malignancy of CRC. HDAC6 is responsible for deacetylating the Lys-116 residue in FOSL1. The initiation of the EMT program in CRC cells can be triggered by the pro-inflammatory cytokine IL-6 stimulation. Further investigation into the mechanism demonstrated that IL-6 induced the transactivation of FOSL1 by activating the phosphorylation and acetylation of STAT3 simultaneously. Clinical sample analyses provided evidence that FOSL1, which is aberrantly induced by IL-6/STAT3, plays a crucial role in mediating the EMT and aggressiveness of CRC. In addition to promoting EMT features, IL-6 induces stem-like properties in colorectal cancer cells, such as the expression of CSC markers CD44+/CD133+, the formation of spheroids, and increased drug-resistance, through FOSL1-dependent pathways. These pathways involve the transactivation of the stemness master gene NANOG, which is mediated by the binding of the K116-deacetylated FOSL1 to the NANOG promoter [92].

In summary, FOSL1 is emerging as a key regulator of CSCs in several cancer types. Their dysregulation contributes to CSC self-renewal, survival, and resistance to therapy. Therefore, targeting FOSL1 and their downstream effectors represents a promising approach to preventing tumor recurrence and improving patient outcomes. T-5224 is a specifically designed small molecule inhibitor that targets the FOS/JUN dimer to prevent transcription [93]. It is now undergoing phase II clinical trials in Japan for the treatment of rheumatoid arthritis [94,95]. T-5224 possesses the capacity to eradicate Bmi1+ CSCs, hence inhibiting the development of HNSCC tumors and overcoming chemoresistance caused by CSCs in a spontaneous mouse model of HNSCC [96]. The direct targeting of the FOS/JUN dimer and suppression of its transcriptional activity by T-5224 is unclear. Given the established knowledge that AP-1 regulates itself through a positive feedback loop, it is necessary to confirm if the removal of HNSCC CSCs by T-5224 is a direct result of reducing FOSL1. 

## 4. FOSL1 Serves as an Independent and Prognostic Factor

FOSL1 holds promise as a prognostic tool, as tumors with increased FOSL1 levels are often associated with aggressive cancer forms. In addition, overexpression of FOSL1 can result in chemotherapy resistance [97]. Mutated KRAS activation is a primary cause of lung cancer deaths with inadequate knowledge of its downstream targets and growth mechanisms of tumor cells. FOSL1 was identified as a crucial factor in regulating the survival and proliferation of human lung adenocarcinoma cells, particularly in the presence of KRAS mutations. Therefore, FOSL1 activation may serve as a potential target for human lung cancer therapy and a prognostic marker, particularly in cases with KRAS mutations [98]. FOSL1/AP-1 transcription factor plays a role in causing lung damage and death in mice when exposed to LPS. FOSL1 achieves this by controlling the expression of proinflammatory cytokine genes through the regulation of c-Jun and NF-kB signaling. This study highlights the potential of targeting FOSL1 to reduce lung injury and inflammation in patients with acute lung injury and adult respiratory distress syndrome, as well as to minimize the impact of endotoxin exposure in clinical settings [99].

FOSL1 ectopic expression was found to be increased in both CRC tissues and cells and was strongly associated with a higher risk of lymph node metastasis, advanced TNM staging, and poorer tumor differentiation. Additionally, there was a demonstrated correlation between FOSL1 ectopic expression and lymphatic invasion, TNM stage, and lower 5-year survival rates in patients with gastric adenocarcinoma [81]. The depletion of FOSL1 results in a significant reduction in tumor burden, by up to 200-fold. Additionally, a FOSL1 classifier was created by comparing RNA profiles of CRC cells that were either parental or FOSL1-depleted. This classifier can predict the prognosis of colon cancer patients. Through functional pathway analysis, it was discovered that the Wnt pathway is central in the classifier, indicating a possible mechanism of how FOSL1 functions in colon cancer metastasis. These findings demonstrate that FOSL1 plays a crucial role in determining the metastatic potential of human colon cancer cells. Moreover, the FOSL1 classifier has the potential to serve as a prognostic predictor for colon cancer patients [100]. 

The significance of FOSL1 as a prognostic factor for patients with ER(+) breast cancer is highlighted, and it is suggested that FOSL1 has distinct roles in breast tumor cells depending on the ER status. In addition to its previously known effects on promoting cell proliferation and invasion, FOSL1 may also impact breast cancer progression by modulating the adhesion of tumor cells to endothelial surfaces [101]. Recent research has shown FOSL1 to be a valuable clinical outcome indicator for breast cancer patients who are ER-negative. The study revealed that patients with higher levels of FOSL1 ectopic expression had shorter overall survival rates, although this correlation was not observed in those with ER-positive breast cancer. Additionally, the study discovered that TNBC cells had higher levels of FOSL1 ectopic expression than ER-positive cells due to more enhancers present in the FOSL1 sequence in TNBC cells. The reliance on copy number alterations (CNAs) in the FOSL1 gene was noticeably higher in the TNBC subtype compared to luminal tumors, indicating a link with more aggressive phenotypes of breast tumors.

The risk of developing oral squamous cell carcinoma (OSCC) is influenced by the transcription factors c-Jun and FOSL1, which also play a significant role in the cancer’s progression. A poor prognosis is linked to the high expression of c-Jun or FOSL1, with FOSL1 ectopic expression resulting in a worse prognosis than c-Jun expression. The interaction between c-Jun and FOSL1 is antagonistic, implying that their activities may impact each other. Both c-Jun and FOSL1 are high-risk predictors of death from OSCC. These findings offer a new perspective on the prognosis and treatment of OSCC [102]. To promote tumorigenicity and metastasis, FOSL1 selectively associates with mediators to establish super-enhancers (SEs) at various cancer stemness and pro-metastatic genes, such as SNAI2 and FOSL1 itself. FOSL1 depletion disrupts SEs and inhibits the expression of these critical oncogenes, leading to tumor initiation and metastasis suppression. Additionally, a positive correlation between FOSL1 and SNAI2 abundance in HNSCC, and high expression levels of FOSL1 and SNAI2 corresponded with shorter overall and disease-free survival. These results demonstrate that FOSL1 functions as a master regulator in promoting HNSCC metastasis through SE-driven transcription and is a promising therapeutic target [13]. Knocking down FOSL1 significantly decreased cell adhesion and migration, while overexpressing an active FOSL1 mutant (FOSL1DD) increased these processes in a JNK/c-JUN-dependent manner. KIND1 was identified as a cytoskeletal regulator of the cell adhesion molecule β1-integrin, as a new transcriptional target of FOSL1. The study also found that recovery of migratory defects that resulted from the loss of FOSL1 was achieved by restoring the expression of KIND1. In line with the in vitro data, HNSCC cells with FOSL1 loss showed decreased rates of subcutaneous tumor growth and pulmonary metastasis. The findings suggest that FOSL1 has a role in promoting cancer growth via AKT and enhancing cancer cell migration through JNK/c-Jun, identifying FOSL1 as a significant integrator of JNK and AKT signaling pathways, and a promising therapeutic target for treating cSCC and HNSCC [103]. 

The outlook for individuals with hepatocellular carcinoma (HCC) is very poor, and there are only a few drugs available that specifically target this type of cancer. Despite the application of new techniques such as high-throughput sequencing, there has been little progress in the identification of prognostic biomarkers for HCC. FOSL1 ectopic expression was analyzed in 20 pairs of fresh HCC tissues and 114 paraffin-embedded HCC tissues. The findings indicated that there is a strong correlation between high FOSL1 ectopic expression and larger tumor size, advanced T stage, and lower survival rates. Elevated levels of FOSL1 are a significant predictor of poor prognosis in HCC patients and serve as an independent risk factor, suggesting that detecting FOSL1 ectopic expression can help identify high-risk patients, and targeting FOSL1 could be a viable therapeutic strategy for HCC treatment [104].

FOSL1 plays a crucial role in maintaining stem cell properties of the MES subtype of GBM, making it an attractive target for therapy. Although inhibiting FOSL1/FOSL1 pharmacologically may be challenging due to its oncogenic function, targeting therapy using techniques such as CRISPR/Cas9 or PROTAC that target FOSL1/FOSL1 ectopic expression could be a promising strategy to treat patients with mesenchymal GBM [46]. In our previous publication, the TCGA database, which contained information on 454 glioblastoma patients, classified as GBM based on the WHO classification, was analyzed for the clinical significance of FOSL1 mRNA expression. FOSL1 mRNA expression levels in GBM patients were significantly higher than those in normal individuals as detected by Affymetrix HT HG U133A; furthermore, classical, mesenchymal, and neural molecular subtypes in glioma tissue were higher than those in the normal brain (*p* < 0.05). Although a similar tendency was displayed in the proneural subtype, it did not reach a significant difference. To determine whether FOSL1 gene expression is related to patient survival, all GBM patients from The Human Protein Atlas were analyzed in a pooled setting; the 7-year overall survival (OS) rate, as revealed by the Kaplan–Meier survival curves, was significantly higher (*p* < 0.001; log-rank test) in those with low FOSL1 transcript levels compared with those with high expression [21]. Recently, several new binding partners of FOSL1were identified in human Th17 cells, which include HDAC2, XRN1, AP2A1, PCBP1, ILF3, TRIM21, and HNRNPH1 [105]. Using Affymetrix HT HG U133A data from TCGA, we observed that TRIM21 is associated with an unfavorable outcome, whereas HDAC2, PCBP1, and HNRNPH1 are favorable factors for glioma patients’ survival.

Taken together, FOSL1 is a prognostic factor in various types of cancer, including breast, lung, liver, and colorectal cancer. Its expression has been associated with tumor progression and poor prognosis, and it has been identified as an independent prognostic factor in several studies. Cancer drug resistance is a key factor in the failure of efficacy treatment with chemotherapy agents. Increased resistance to chemotherapy agents in cancer cells has several causes, including CSC, multidrug resistance transporter 1(MDR1), defects in DNA repair, ABC transporters, and the elevated expression of anti-apoptotic proteins [106,107,108]. Past studies report that transcription factors such as FOSL1 have a pivotal role in resistance to chemotherapy agents [107]. There are several reasons why FOSL1 can serve as a useful prognostic tool. Firstly, solid tumors with a higher grade usually show intense staining for FOSL1. Secondly, aggressive forms of cancer are often associated with tumors that have higher levels of FOSL1. Lastly, increased expression of FOSL1 has been linked to resistance to chemotherapy. By monitoring the levels of FOSL1, it could be possible to determine the grade of the tumor [109].

## 5. The Role of FOSL1 in Drug Resistance 

It has been reported that the expression levels of FOSL1 in drug-resistant breast cancer cells, specifically MCF-7/ADR and MDA-MB-231/ADR, noticeably increase, leading to the induction of resistance to doxorubicin both in vitro and in vivo. It was revealed that FOSL1 promotes the expression of dual specificity phosphatase 7 (DUSP7) via binding to its promoter. Subsequently, through DUSP7, FOSL1 induces dephosphorylation of PEA15 (proliferation and apoptosis adaptor protein 15). Dephosphorylated PEA15 promotes cell proliferation, invasion, and migration while inhibiting apoptosis. Taken together, the FOSL1/DUSP7/DEA15 axis promotes drug resistance in breast cancer [110]. In another study, it was reported that breast cancer patients who were treated with epirubicin-based neoadjuvant chemotherapy (NCT) showed a significant suppression in the expression levels of FOSL1. Duan et al., reported that the levels of FOSL1 in NCT-sensitive patients were significantly lower than those in NCT-resistant patients. Knockdown of FOSL1 in the MCF-7/ADR cancerous cell line sensitized the cells to doxorubicin, resulting in decreased cell proliferation and cell growth [111]. Dan et al., paradoxically reported that there is a negative correlation between the expression of FOSL1 and clinical chemoresistance in breast cancer patients. They indicated that the recurrence of the tumor is delayed when the expression of FOSL1 is increased. They demonstrated that silencing FOSL1 ectopic expression led to a significant increase in the population of CSCs, while ectopic expression of FOSL1 resulted in a decrease in the CSC population. Both in vitro and in vivo data revealed that FOSL1 enhances chemosensitivity to doxorubicin and cyclophosphamide by driving CSCs from dormancy [112].

Wu et al. reveal that the axis involving FOSL1–miR-134–SDS22-JNK/ERK enhances drug resistance in ovarian cancer cells. They demonstrated that FOSL1 induces miR-134 expression while suppressing SDS22 expression, as SDS22 is one of the targets of miR-134. Consequently, this leads to an increase in the activity of JNK/ERK signaling pathway, which further promotes FOSL1 ectopic expression. Taken together it can be concluded that the FOSL1–miR-134 axis drives a positive feedback loop, promoting ERK/JNK signaling and enhancing chemoresistance in ovarian cancer cells [113].

Cancer-associated fibroblasts (CAFs) constitute a significant portion of cells present in the TEM. Lin and Zhu reported that exosomes derived from CAFs isolated from CRC stromal cells are enriched with FOSL1. Upon transferring these to CRC cells, they observed a significant increase in stemness, cell proliferation, and resistance to oxaliplatin. FOSL1 facilitates these effects by promoting integrin β4 (ITGB4) transcription [114]. 

After subjecting colorectal cancer cell lines (SW480 and SW620) to X-ray or c-ion radiation, the expression level of FOSL1 remarkably decreases. Endo et al. reported that the sensitivity of cancerous cells to radiation increases after the knockdown of FOSL1 [115]. In pancreatic ductal adenocarcinoma (PDAC) cell lines, Bosutinib, a small molecule inhibitor, when used alone and in combination with chemotherapy agent (5′-fluorouracil and gemcitabine), decreases the proliferation and tumorigenesis of cancerous cells. Bosutinib sensitizes cancer cell lines to 5′-fluorouracil and gemcitabine by disrupting the Src-FOSL1 axis, resulting in decreased expression of mucins (MUC4 and MUC5AC) [116].

**Figure 4:**
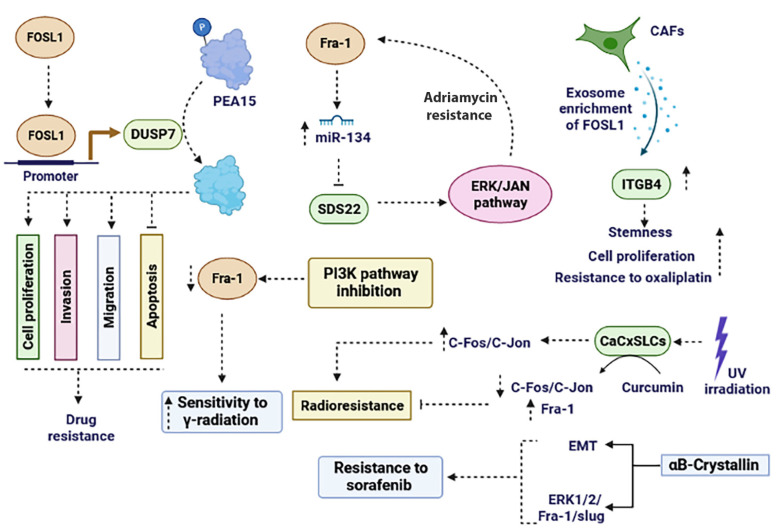
The role of FOSL1 in promoting drug resistance and radio resistance. FOSL1 plays a role in drug resistance through various mechanisms. It enhances resistance to chemotherapy agents and radiotherapy by influencing the PI3K, ERK, ERK/JAN, and ITGB4 signaling pathways. Moreover, FOSL1 exerts a critical role in cancer drug resistance by regulating processes like EMT, apoptosis, invasion, migration, and miRNAs. EMT: epithelial–mesenchymal transition.

In HCC cancer cells, the ectopic expression of αB-Crystallin promotes resistance to sorafenib by inducing EMT and subsequently activating the ERK1/2/FOSL1/slug signaling pathway [117]. Inhibition of the PI3K signaling pathway decreases the expression of FOSL1 as an AP-1 subunit and enhances sensitivity to γ-radiation in prostate cancer cells [118]. In cervical cancer stem-like cells (CaCxSLCs), there was an increased expression of AP-1 family members (c-Fos, c-Jun, Jun B, and Jun D) at both proteins and mRNA levels, but the FOSL1 transcript was lacking. Upon exposure of CaCxSLCs to UV-irradiation, the expression level of c-Fos and c-Jun remarkably increased, resulting in radio resistance. However, pre-treatment of CaCxSLCs with curcumin followed by UV-irradiation led to the suppression of c-Fos and c-Jun while upregulating FOSL1. Targeting the AP-1 family sensitized CaCxSLCs to UV-irradiation by increasing FOSL1 [108] (Figure 4). In summary, targeting FOSL1 has emerged as a potential strategy for overcoming drug resistance in cancer. Its overexpression is associated with poor prognosis and resistance to chemotherapy and targeted therapies.

FOSL1 has emerged as a potential contributor to GBM resistance against erlotinib, an EGFR tyrosine kinase inhibitor. This identification suggests that targeting FOSL1 could enhance the effectiveness of erlotinib in combating GBMs [45]. When both EGFR and MET were inhibited, the transient suppression of FOSL1 phosphorylation was observed, followed by a rebound effect, leading to elevated transcription levels of five FOSL1 target genes [48,49,50,51,52,53,54]: SPRY2, MMP2, JUN, PLAUR, MMP1, and IL-6. This cascade contributed to resistance against kinase inhibitors. Our unpublished data showed that silencing FOSL1 potentiates the GBM cell response to TMZ. Having established that FOSL1 signaling is active in GBM cell models and contributes to cell proliferation, we then asked whether FOSL1 activity plays a role in cellular resistance to TMZ. To test this, U87MG (sensitive to TMZ) and T98G cells (resistant to TMZ) were transiently transfected with siFOSL1 or siCtrl with a range of TMZ doses (0–500 µM). Treatment efficacy was assessed using an MTT assay to measure cell viability 72 h after transfection. A dose–response experiment showed TMZ produced an additive decrease in cell viability with siFOSL1 for all TMZ doses tested in TMZ-sensitive U87MG cells. In TMZ-resistant T98G cells, siFOSL1 alone had a strong inhibitory effect, while the addition of TMZ had a more inhibitory effect. A further 4-day time course experiment of T98G cells treated with the highest dose of TMZ (500 µM) confirmed additive effects on cell proliferation in a time-dependent pattern. 

## 6. Conclusions

FOSL1, a transcription factor from the activator protein-1 (AP-1) superfamily, plays a significant role in various cancers by promoting tumor growth, survival, differentiation, and invasion. Its expression level is high in many cancer cells and correlates with the severity of the disease and patient survival rates. FOSL1 has been implicated in the development and progression of several types of cancer, including breast, lung, prostate, gastric, colorectal cancer, and glioma. It exerts its oncogenic effects by regulating various signaling pathways, including MAPK, PI3K/Akt, Wnt/β-catenin, and TGF-β, which are involved in cell proliferation, survival, apoptosis, and metastasis. Recently, it has been revealed that noncoding RNAs (ncRNAs) play important roles in the regulation of FOSL1 expression and function. Additionally, FOSL1 has been found to modulate the expression of various cytokines, chemokines, and extracellular matrix (ECM) proteins that hold crucial functions in the TME. For example, FOSL1 has been reported to upregulate the expression of IL-6 in breast cancer cells, which can promote tumor growth and invasion by activating the STAT3 signaling pathway. FOSL1 has also been shown to play a role in cancer stemness. CSCs are a subpopulation of tumor cells that are capable of self-renewal, differentiation, and tumor initiation. FOSL1 is overexpressed in CSCs of various cancers, including breast, colorectal, and lung cancers. The inhibition of FOSL1 in colorectal cancer cells has been found to reduce the population of cancer stem cells and inhibit tumor growth. There is growing evidence suggesting that FOSL1 may contribute to drug resistance in cancer cells. One study found that the overexpression of FOSL1 in breast cancer cells led to increased resistance to the chemotherapy drug doxorubicin, while the downregulation of FOSL1 increased sensitivity to the drug. Out of all the members of AP-1, FOSL1 has been proven to play a crucial role in supporting the malignant progression of malignancies. Targeting FOSL1 has a strong inhibitory effect on the formation and spread of specific types of cancers. Despite extensive endeavors, no drugs targeting AP-1 or FOSL1 for cancer treatment have been approved for clinical use. The limited understanding of the structure and function of FOSL1 hinders the development of strategies to target FOSL1 with small molecule inhibitors for tumor treatment. Hence, it is imperative to implement innovative approaches and conduct additional verifications. 

## Figures and Tables

**Table 1:** The role of FOSL1 in different cancers.

Cancer Type	Clinical Samples	Assessed Cell Line	FOSL1 Expression	Effects: In Vitro/In Vivo	Regulatory Mechanism	Ref.
BC	51 paired tissues	MDA-MB-231, Hs578T	Up	∆ miR34a/c: ↓ Invasion, ↓ Migration	miR-34a/c-Fra1	[30]
	30 paired tissues	MDA-MB-231, MCF7, BT-474, SK-BR-3, BT-20, NF, CAF	UP	∆ miR4516: ↓ Proliferation	miR4516/FOSL1	[31]
	33 TNBC and 34 non-TNBC patients	MDA-MB-231, Hs578T	UP	∆ miR-130a: ↑ Tight-junction protein, ↓ Invasion, ↓ Migration	miR-130a/FOSL1/ZO1	[29]
	-	4T1, 4T07, RAW264.7	UP	∆ miR-19a-3p: ↓ Metastasis, ↓ Invasion, ↓ Migration, ↑ Macrophage polarization, ↓ Tumor growth	miR-19a-3p/Fra-1/STAT3	[32]
		MCF7, 4T1, ZR-75-1	UP	∆ FOSL1: Invasion ↓, Migration ↓	MLK3–FRA-1–MMP-1	[56]
		MDA-MB-231, BT549	UP	∆ FOSL1: Invasion ↓, Migration ↓	ERK1/2, SPAK	[57]
		MDA-MB-231, MDA-MB-436, MCF-7	UP	∆ FOSL1: Invasion ↓, Migration ↓	HMGA1	[58]
	fourth node mice	BRC-17, BRC-31, BRC-32, BRC-36, BRC-	UP	∆ FOSL1: Invasion ↓, Migration ↓	Integrin-uPAR	[58]
		4T1	UP	∆ FOSL1: Invasion ↓, Migration ↓	IL-6/JAK/Stat3	[59]
CRC	-	CRL-1831, LoVo, RKO, HCT15, HCT28, HCT116, and SW480	UP	∆ miR-497: ↓ EMT, ↓ Migration, ↓ Invasion	miR-497/Fra-1	[34]
	40 paired tissues	HEK293T, RKO, HCT116, HCT116	UP	▼Fra-1 or ∆ miR-34a: ↓ Migration, ↓ Invasion	miR-34a/p53/Fra-1	[33]
		HCT116	UP	∆ FOSL1: Invasion ↓, Migration	miR-34a/Fra1	
		HT-29, HCT116	UP	∆ FOSL1: Invasion ↓, Migration ↓	USP21	[60]
		SW620, HCT116, DLD1 HT29	UP	∆ FOSL1: Invasion ↓, Migration ↓	RAS	[61]
	paired tissues, PDX mouse model		UP	∆ FOSL1: Proliferation ↓, Invasion ↓, Migration ↓,	EGFRMAPK-FRA-1	[62]
GC	20 paired tissues	AGS	UP	∆ FOSL1: Proliferation ↓, apoptosis	PI3K/Akt, p53	[63]
NPC	20 NPC and 16 non-NPC patients	NP69, N2-Tert, C666-1, CNE1, CNE2, HK1, HNE1, HONE1, SUNE1, 5–8F and 6–10B	UP	▼LINC01503: ↓ Proliferation, ↓ Invasion, ↓ Migration, ↓ Metastasis, ↓ Tumor growth	LINC01503/SFPQ/FOSL1	[24]
	53 paired tissues	CNE1	UP	∆ FOSL1: Proliferation ↓, Growth tumors ↓	MSK1, LMP1	[64]
LC	55 paired tissues	H460	UP	∆ FOSL1: apoptosis ↓	p53	[65]
OS		Saos2, MG63	UP	∆ FOSL1: Proliferation ↓, Invasion ↓, Migration ↓,	ERK/AP-1	[66]
GBC	-	NOZ, GBC-SD	UP	∆ miR-195-5p: ↓ Proliferation, ↑ G0/G1 phase, ↓ Invasion, ↓ Migration, ↓ Tumor growth	miR-195-5p/FOSL1/Wnt pathway	[28]
TC		FRTL-5^K-Ra^	UP	∆ FOSL1: Proliferation ↓	cyclin A, Jun B	[64]
	paired tissues	BHP10-3, BCPAP, SNU790	UP	∆ FOSL1: Invasion ↓, Migration ↓	ERK/Fra-1/ZEB1	[67]
HSCC	40 paired tissues	FaDu, Detroit 562, NP69	UP	▼HOXA11-AS1: ↓ Immune escape, ↓ Metastasis, ↓ Proliferation, ↓ Tumor growth	HOXA11-AS1/FOSL1/PTBP1/PD-L1	[36]
EC	53 paired tissues	E70, KYSE-510, EC9706, HEECs	UP	▼AGAP2-AS1: ↓ Proliferation, ↓ Invasion, ↓ Migration, ↑ Apoptosis, ↑ G0/G1 phase, ↓ Tumor growth	AGAP2-AS1/miR-195-5p/FOSL1	[25]
Glioma	-	A172, T98G, U87MG	UP	▼FOSL1: ↑ Apoptosis, ↓ Tumor growth	FOSL1/miR-301a-3p	[26]
	22	U251	UP		miR-33a/FOSL1	[55]

(∆: over-expression, ▼: knockdown), up arrow: increased. down arrow: decreased. BC: breast cancer, CRC: colorectal cancer, GBC: gallbladder carcinoma, HSCC: hypopharyngeal squamous cell carcinoma, NPC: nasopharyngeal carcinoma, EC: esophageal cancer, GBM: glioblastoma, HNSCC: head and neck squamous cell carcinoma, TNBC: triple-negative breast cancer, TC: thyroid cancer, OS: osteosarcoma, LC: lung cancer, GC: gastric cancer.

## Data Availability

Data will be made available on reasonable request.
